# Neural signatures for sustaining object representations attributed to others in preverbal human infants

**DOI:** 10.1098/rspb.2015.1683

**Published:** 2015-11-22

**Authors:** Dora Kampis, Eugenio Parise, Gergely Csibra, Ágnes Melinda Kovács

**Affiliations:** 1 Department of Cognitive Science, Central European University, Budapest 1051, Hungary; 2 Department of Psychology, Flyde College, Lancaster University, Lancaster LA1 4YF, UK; 3 Department of Psychological Sciences, Birkbeck, University of London, London WC1E 7HX, UK

**Keywords:** infant cognitive development, social cognition, object representation, theory of mind, metarepresentation

## Abstract

A major feat of social beings is to encode what their conspecifics see, know or believe. While various non-human animals show precursors of these abilities, humans perform uniquely sophisticated inferences about other people's mental states. However, it is still unclear how these possibly human-specific capacities develop and whether preverbal infants, similarly to adults, form representations of other agents' mental states, specifically metarepresentations. We explored the neurocognitive bases of eight-month-olds' ability to encode the world from another person's perspective, using gamma-band electroencephalographic activity over the temporal lobes, an established neural signature for sustained object representation after occlusion. We observed such gamma-band activity when an object was occluded from the infants' perspective, as well as when it was occluded only from the other person (study 1), and also when subsequently the object disappeared, but the person falsely believed the object to be present (study 2). These findings suggest that the cognitive systems involved in representing the world from infants' own perspective are also recruited for encoding others' beliefs. Such results point to an early-developing, powerful apparatus suitable to deal with multiple concurrent representations, and suggest that infants can have a metarepresentational understanding of other minds even before the onset of language.

## 1. Introduction

Humans and other animals encode various aspects of the world, allowing them to successfully navigate their physical and social environment. What possibly sets humans apart from other species is that they attribute mental states to other people as representations of the environment that may be different from their own representations. This ability is usually termed as theory of mind and includes reasoning about others' mental states such as beliefs, goals or desires [1]. Theory of mind capacities seem to emerge early in human development, and a growing body of evidence suggests that infants can interpret others' behaviour with regard to their mental states very early on [2,3], although the underlying mechanisms are still unclear.

Already in their first year of life, infants can predict others' actions based on their mental states [4], and at around 18 months of age, they can also modify their own behaviour accordingly [5–7]. Such findings were taken as evidence that infants attribute beliefs to others and represent these belief contents in the form of metarepresentations (i.e. representations incorporating other representations) [8]. Other accounts, however, question the validity of the interpretation of these studies in terms of mental state attributions, and suggest that instead of ascribing mental representations to others, infants simply store object–agent relations [9], form associations or apply behavioural rules [10]. Similar alternatives were also raised with regard to non-human animals' theory of mind abilities [11]. Metarepresentations in general, and theory of mind or false belief understanding in particular, have been argued to be absent in other species than humans [12–14]. Thus, to understand the nature and origins of such abilities, it would be crucial to assess whether prelinguistic creatures, specifically human infants attribute representations to other people.

Different accounts emerged regarding how humans may deal with metarepresentations used in language and in mental state reasoning. Sperber [15] proposed that the most cost-effective way for a cognitive system to handle them would be if *any* representation could also serve as the content of a metarepresentation. Leslie [8], in his work describing a cognitive model of pretense (make-believe play observed in toddlers, such as pretending that a banana is a telephone), argues that in pretense the primary representation of an object is copied into a ‘metarepresentational context’. Both these proposals involve some form of ‘re-use’ of a primary representation. The relation between a linguistic utterance as a primary representation (e.g. ‘dogs can fly’) and a corresponding metarepresentation (e.g. it is unlikely that ‘dogs can fly’) may seem intuitive. However, it is unclear how such embedded representations are implemented in the cognitive architecture, how they are realized in case of non-linguistic representations, and whether the underlying mechanism could be already present in preverbal infants.

Earlier research from the domain of action understanding might provide useful insights regarding how the developing cognitive system may deal with representations that regard other people. Specifically, recent electrophysiological findings suggest that infants recruit their motor system (as reflected by decreased sensorimotor alpha-band oscillatory activation) not only when they perform an action, but also during the observation and prediction of others' actions [16]. In the domain of theory of mind, behavioural evidence seems to point to an analogous possibility. When infants or adults are exposed to situations where they can track others' perspective or beliefs, their own representations and the representations attributed to others seem to influence their reactions in analogous ways [3,17]. For example, infants show surprise when the outcome of an event does not match another agent's belief about the scene, similarly to their surprise if the outcome contradicts their own knowledge [3]. These findings suggest that the two representations may overlap, and are in line with the proposal that a possible mechanism for infants (or adults) to attribute representations to others would use their own representational system that is otherwise used for encoding objects and events in the world.

In this study, we build on this proposal: if infants ascribe a representation to another person, say, about an object, they would rely on their original representation, which would then be used as the content of the mental state. This way infants' own representations of the environment and the representations ascribed to others could be realized through one cognitive system subserving both processes. If so, this enables us to make predictions about the neural signatures of processing ascribed representations. For example, if maintaining a representation of an object, as a primary representation, has a specific neural correlate in infants, we should observe a similar neural activation also if infants process an object representation they attribute to another person. To test these questions, we exploit earlier paradigms that found a specific brain signature accompanying object representations in infants.

Infants possess powerful representational abilities to sustain the representation of an object even if it is not visible to them anymore. Kaufman *et al.* [18] found increased gamma-band oscillatory activation in electroencephalographic (EEG) responses over the temporal regions when six-month-old infants witnessed the occlusion of an object, compared with when the object disintegrated before occlusion. Similar activation was found when a hand began lifting an occluder behind which an object had previously entered [19]. Together, these findings suggest that the gamma-band activation signalled that infants actively sustained the representation of the object, which they believed to be behind the occluder. Here, we hypothesize that such activation may not only reflect processes involved in how infants handle object representations for themselves, but also signal computations required for attributing a representation about an object to another person.

In two studies, we presented eight-month-old infants with scenes involving an actor and an object, and recorded event-related EEG activity during events involving the occlusion of the object from the infants' or the actor's perspective. An increase in gamma-band activation was predicted when either the infant or the actor had to sustain the representation of the object.

## 2. Study 1

**Figure 1.**
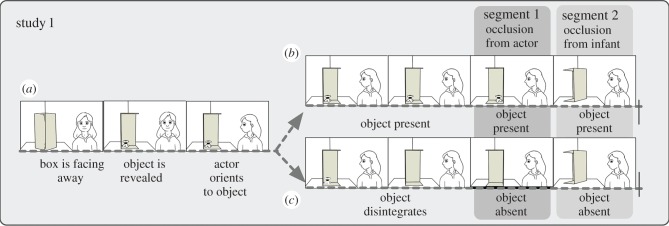
Schematic of the events in study 1. (*a*) The first 1.5 s of each video were identical in the two conditions. (*b*) In the object present—occlusion condition the object remained present, and was occluded by the rotating box, first from the actor's point of view, then also from the infant. (*c*) In the object absent—occlusion condition, the rotation of the box was identical but occluded an empty area from the actor's and then the infant's side. In both conditions, the trial ended with the box completely turned away.

Study 1 explored eight-month-old infants' understanding of a scene where a person is attending to an object, which is then occluded from her. We asked whether this event triggers an attribution process that involves sustained object representations. In order to test this, we developed scenarios involving occlusion events from multiple perspectives (figure 1). First, a target object and an actor were shown on the screen, with the object visible to both the infant and the actor. Then, the object was occluded either from only the actor or also from the infant's view. In order to implement a dynamically changing visual access to the object from multiple viewpoints, we placed the object in a box that had two sides removed. By rotating the box, the infant, the actor on the screen, neither or both could see the object in question. We compared these events with scenarios where the box initially contained an object, but then the object disintegrated while both the actor and the infants could see this event. Therefore, the motion of the box was identical in the two kinds of events, but in this latter case, the box did not occlude an object from the actor's or infant's view (rather just empty space).

On the basis of previous findings [18,19], we predicted increased gamma-band activation during the occlusion of the object from the infants' view. Furthermore, we hypothesized shared underlying mechanisms for sustaining an object representation for the self and for another person. Therefore, increased gamma-band oscillatory activity during occlusion from the actor would suggest that infants encode that the actor sustains the representation of the object while it is occluded from her. We calculated the average EEG gamma-band activation (25–35 Hz) over the left and right posterior temporal regions specified by earlier studies targeting sustained object representations in infancy [18,19], during occlusion of the object from the actor's or the infants' view.

### (a) Material and methods

#### (i) Participants

The final sample consisted of 15 full-term eight-month-old infants (mean age = 246.3 days; age range 236–255 days).

#### (ii) Stimuli

Two types of videos were used (corresponding to two conditions). Both featured a female actor who looked at a rotating box open at two sides that contained an object. First, the opening of the box was facing away for 200 ms, then it rotated to reveal the object in 600 ms and stood still for 200 ms. Then, the actor turned to the object for 600 ms. This was followed by the object remaining present (object present—occlusion condition; electronic supplementary material, video S1) or the object disintegrating in 600 ms (object absent—occlusion condition; electronic supplementary material, video S2). Following a 300–500 ms (randomized length) still period, the box turned further, occluding the object (object present—occlusion condition) or an empty area (object absent—occlusion condition) from the actor in 600 ms. After a 700–900 ms (randomized length) still period, the box rotated again further and occluded the object (object present—occlusion condition) or an empty area (object absent—occlusion condition) also from the infant. The trial ended with an 800 ms still period with the box completely turned away (identical in the two conditions). For further details regarding stimuli and procedure, see electronic supplementary material, materials and methods.

#### (iii) EEG recording and analysis

Continuous EEG was recorded using Hydrocel geodesic sensor nets (Electrical Geodesics Inc., Eugene, OR) from 124 channels equally distributed on the scalp, referenced to the vertex (Cz). The ground electrode was at the rear of the head (between Cz and Pz). The sampling rate was 500 Hz with a low-pass filter of 200 Hz. The EEG was segmented into two types of segments of interest.

The first segment (occlusion from actor) was the part of the video when, in the object present condition, the object was gradually hidden from the actor due to the rotation of the box, while the infants still saw it. In the object absent condition, this segment included the identical movement of the empty box. This segment was time-locked to the start of the movement of the box, and lasted 1200 ms after rotation onset, of which the rotation took place in the first 600 ms. The baseline period for the occlusion from actor segment was the 200 ms recording preceding the rotation of the occluder. The second segment of interest (occlusion from infant) corresponded to the period when the object became gradually hidden from the infants. This segment was time-locked to the start of the respective movement of the occluder and had a length of 1200 ms. In the occlusion from infant segment, we used an epoch that roughly matched (due to a jittered period after the occlusion from actor segment), the baseline period in the first segment: a 200 ms interval ending 1500 ms before the onset of occlusion from infant (for calculating this baseline period, see electronic supplementary material, material and methods).

The EEG data were examined, and segments were excluded if they were judged as not attended by the infant based on the video recording, or contained artefacts as judged by the automatic or manual artefact detection (for further details, see electronic supplementary material, materials and methods). After the time–frequency transformation performed on the cleaned data, we compared oscillatory activity between the two conditions over 5–5 channels in right (channels 97, 98, 102, 103, 109, positioned above channel T3 in the 10–20 system) and left (channels 40, 41, 46, 47, 51, above channel T4 in the 10–20 system) temporal areas. Electrode sites were selected based on previous work by Kaufman *et al.* [18,19]. We analysed the lower frequencies (25–35 Hz) of the gamma range, where activation was most pronounced in earlier studies [19] for our events of interest.

### (b) Results

**Figure 2.**
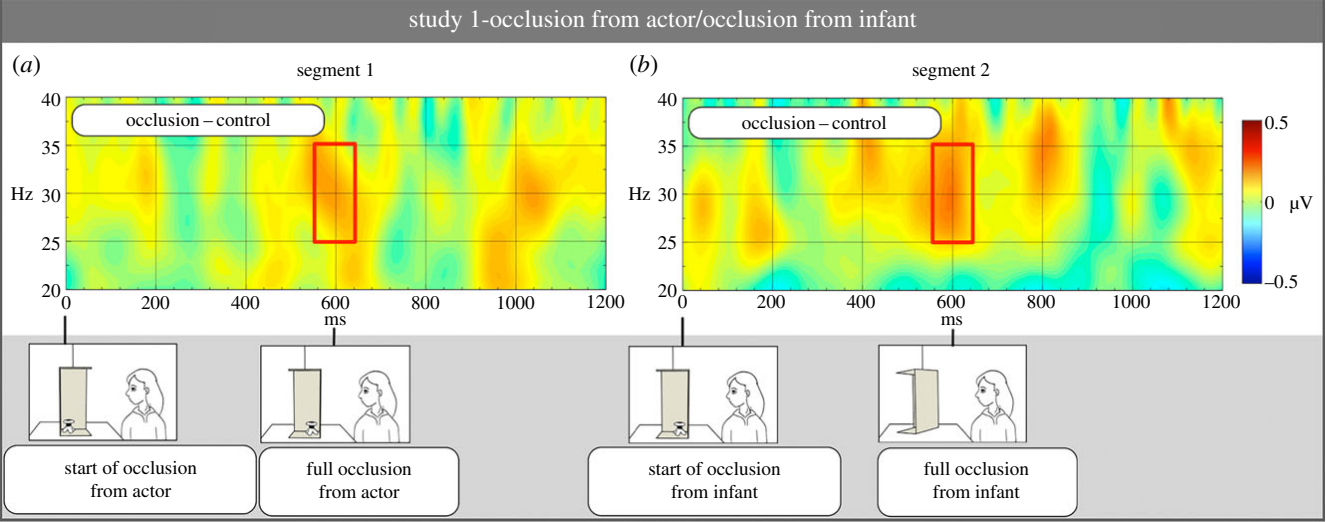
Time–frequency difference plots depicting average gamma-band oscillatory activation over the left and right posterior temporal cortex during the two segments in study 1. Plots reflect mean activation difference between conditions; positive difference indicates higher activation in object present—occlusion condition than in object absent—occlusion condition. In both segments, 0 ms marks the onset of the occlusion event; in the first segment (*a*) from the actor, in the second segment (*b*) from the infant. Red rectangles indicate the time and frequency range over which statistics were computed.

First, we analysed gamma-band oscillatory activation in the two segments separately, in two-way ANOVAs withcondition (object present—occlusion versus object absent—occlusion) and hemisphere (left versus right) as within-subjects factors. To assess whether our results replicate earlier findings on neural signatures of sustained object representations, we analysed activation during the occlusion from infant segment (segment 2). Analysis revealed a significant main effect of condition (*F*_1,14_ = 13.23, *p* = 0.003, partial *η*^2^ = 0.49) due to significantly higher activation in the occlusion (*M* *=* 0.09 µV, s.e. = 0.03), compared with object absent—occlusion condition (*M* *=* −0.07 µV, s.e. *=* 0.04 µV; figure 2*b*). There was no main effect of hemisphere, and no interaction between condition and hemisphere (*F*_1,14_ = 0.04, *p* = 0.81; and *F*_1,14_ = 0.06, *p* = 0.86).

We conducted a similar two-way ANOVA for the occlusion from actor segment (segment 1), which revealed a significant interaction between condition and hemisphere (*F*_1,14_ = 4.99, *p* = 0.04, partial *η*^2^ = 0.26), and a marginally significant main effect of condition (*F*_1,14_ = 4.53, *p* = 0.052, partial *η*^2^ = 0.24). There was no effect of hemisphere (*F*_1,14_ = 0.06, *p* = 0.81). To understand the interaction, we performed separate *t*-tests for the two hemispheres. There was no significant difference between conditions in the right hemisphere, *t*_14_ = −1.03, *p* = 0.32. Importantly, there was a significant difference in the left hemisphere (*t*_14_ = −2.56, *p* = 0.023, *r*^2^ = 0.32) due to higher gamma activation in the object present—occlusion condition (*M* *=* 0.08 µV, s.e. *=* 0.05 µV) than in the object absent—occlusion condition (*M* *=* *−*0.12 µV, s.e. *=* 0.04 µV; figure 2*a*).

**Figure 3.**
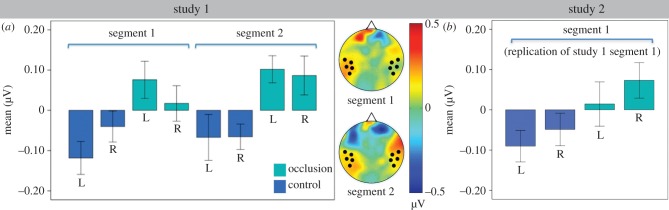
Mean activation in (*a*) study 1 during occlusion from actor and occlusion from infant, and (*b*) study 2 occlusion from actor at the target time windows (550–650 ms), at five left (L) and five right (R) temporal electrodes in object present—occlusion (‘occlusion’) and object absent—occlusion (‘control’) conditions, over the 25–35 Hz frequency range. Error bars represent standard errors.

To assess whether the pattern of activation in the two segments was similar to each other, we analysed them together in a repeated measure ANOVA with segment (occlusion from actor versus occlusion from infant), condition (object present—occlusion versus object absent—occlusion) and hemisphere (left versus right) as within-subjects factors. We found a significant main effect of condition (*F*_1,14_ = 13.24, *p* = 0.003, partial *η*^2^ = 0.49). No other main effect or interaction was significant (for mean values in study 1, see figure 3*a*). Thus, while in the occlusion from actor segment, the effect was more pronounced on the left side, the direction of activation in this segment was similar in the two hemispheres and together they did not differ significantly from that in the occlusion from infant segment.

In addition to analysing activation in our predicted time windows, we observed a further activation within the same frequency range in the occlusion from actor segment for the 1000–1100 ms period (figure 2*a*). When we analysed activation in this additional time window, we found a marginally significant effect of condition in the left hemisphere (*t*_14_ = −2.07, *p* = 0.057, *r*^2^ = 0.23) with higher activation in the object present—occlusion condition than in the object absent—occlusion condition (*M* *=* 0.09 µV, s.e. *=* 0.05 µV and *M* *=* −0.11 µV, s.e. = 0.07 µV, respectively). A similar analysis did not yield any significant effects in the right hemisphere and in the occlusion from infant segment. While this late activation burst in occlusion from actor segment may signal a possible difference between processing representations attributed to another agent and first person representations, it was not predicted. We intended to confirm this finding in study 2.

### (c) Discussion

Our results from the occlusion from infant segment are in line with earlier evidence pointing to a signature of infants' sustained object representation [18,19]. Specifically, we observed higher gamma-band activation over posterior temporal areas when an object became occluded from the infants compared with when there was no object present. Crucially, we observed similar activation when the object became occluded from the actor only (occlusion from actor). Note that in the occlusion from actor segment the object was still visible to infants; therefore, they did not have to sustain the object representation from their own perspective. This suggests that infants attributed a sustained representation of the object to the actor when she lost visual access to the object.

These results suggest that eight-month-old infants successfully computed the visual perspective of the actor regarding the object, an ability that is rarely observed at such a young age. Furthermore, while visual perspective taking (computing whether an agent can see an object) is necessary, it may not be sufficient to explain our findings. Taking the gamma-band oscillatory activity at the time of occlusion as an indicator of sustained object representation, infants in our study did not only infer that the person no longer saw the object (as this would also apply in the object absent-occlusion condition), they also attributed to her the representation of the continued existence of the object behind the occluder.

Identifying the mechanisms at play when infants attribute a sustained object representation (a *true* belief) to another person allows further investigations of belief attribution processes. If the activation found in study 1 accompanies events involving attributed object representations, then it should be present regardless of the veridicality of this representation (i.e. even when the other person holds a *false* belief regarding the object's existence behind the occluder).

## 3. Study 2

We developed a false belief scenario similar to the events in study 1 (see electronic supplementary material, figure S1). Eight-month-old infants were presented with the same initial event in which the actor attended to an object. Then, in the critical condition, the object became occluded from the person (segment 1; identical to segment 1 of study 1), and afterwards, the object disintegrated (segment 2). This disintegration was therefore visible to the infants but not to the actor; hence, this event must have resulted in the actor's false belief that the object was still behind the occluder. The critical question was whether infants would encode that the representation of the object cannot be discarded on behalf of the actor but it must be further sustained. Such an attribution process might be indicated by gamma-band activation during the disintegration event that is seen only by the infant but not the actor.

### (a) Material and methods

#### (i) Participants

The final sample consisted of 15 full-term eight-month-old infants (mean age = 245 days; range = 229–261 days).

#### (ii) Stimuli

In study 2, the setting of the scenes and the initial part of the videos (including the first segment of interest) was identical to study 1. Then, in the object occluded—false belief condition, the object was occluded from the actor by the rotating box in 600 ms (occlusion from actor) and after a still period of 600 ms, it disintegrated during 600 ms, while only the infants and not the actor could see this event (electronic supplementary material, video S3). We will refer to this disintegration period as a false belief event, because in this case infants could note that the object ceased to exist and was not present anymore, and could infer that the actor should falsely believe it still to be present behind the occluding side of the box. In the object absent—true belief condition (electronic supplementary material, video S4), the object disintegrated when the actor still saw the object, and subsequently, the empty space was occluded in 600 ms. Following a 600 ms still period (during the corresponding disintegration period of the object occluded—false belief condition), in the object absent—true belief condition, the empty box remained turned away from the actor for 600 ms. Thus, the two conditions differed only in the timing of the disintegration of the object: after (false belief) or before (true belief), it was occluded from the actor. Finally, in both conditions, the empty box rotated back towards the actor. Hence, infants in study 2 never saw the object being occluded from them. The rotation of the box was identical in the two conditions. For further details regarding stimuli and procedure, see electronic supplementary material, materials and methods.

#### (iii) EEG recording and analysis

Except for segmentation, EEG recording and analysis was identical to that of study 1. Similarly to study 1, the first segment (occlusion from actor) was the part of the video when the object was gradually hidden from the actor by the rotation of the box (in the object occluded—false belief condition), whereas the infants still saw it; or the identical movement of the empty box (in the object absent—true belief condition). Hence, in the occlusion from actor segment, we specified the same time window of interest as in study 1, and the baseline was again a 200 ms interval finishing 1200 ms before the start of the segment.

The second segment of interest (false belief event) in study 2 corresponded to the period when the object disintegrated after being occluded from the person (or the same time period during the object absent—true belief condition) and the subsequent still image. This period lasted 800 ms and its start was time-locked to the start of disintegration event. Similar to study 1, the baseline was a 200 ms interval finishing 1200 ms before the start of the segment (the same baseline as for occlusion from actor). In this false belief segment, we analysed activation throughout the disintegration event, from 1200 to 1800 ms, where 1200 ms corresponded to the onset of the disintegration and 1800 ms to the time point when the object had fully disappeared.

### (b) Results

We calculated the average gamma-band activation (25–35 Hz) the same way as in study 1 during two segments of interest: occlusion from actor and false belief.

As direct comparison between the two segments was not meaningful (one being an occlusion, which can be seen as a discrete event, while the other is a disintegration with a gradual temporal unfolding), activations in the two segments were analysed separately. A two-way ANOVA on the occlusion from actor segment with condition (object occluded—false belief versus object absent—true belief) and hemisphere (left versus right) as within-subjects factors revealed a main effect of condition (*F*_1,14_ = 5.98, *p* = 0.03, partial *η*^2^ = 0.3). This effect was due to higher activation in the object occluded—false belief condition (*M* = 0.044 µV) than in object absent—true belief (*M* = −0.07 µV; figure 3*b*). No other main effect or interaction emerged.

We then compared activation during occlusion from actor in study 2 with that of study 1. These segments were identical in the two studies and both depicted an occlusion from actor event. A three-way mixed ANOVA was conducted with condition (object present versus object absent) and hemisphere (left versus right) as within-subjects factors, and study (1 versus 2) as a between-subjects factor. This analysis revealed a main effect of condition (*F*_1,28_ = 10.13, *p* = 0.004, partial *η*^2^ = 0.27), which was due to higher activation in the object present condition (*M* *=* 0.05 µV, s.e. *=* 0.03 µV) than in object absent condition (*M* *=* −0.07 µV, s.e. *=* 0.02 µV). There was no effect of study (*F*_1,14_ = 0.01, *p* = 0.92), and no interaction.

**Figure 4.**
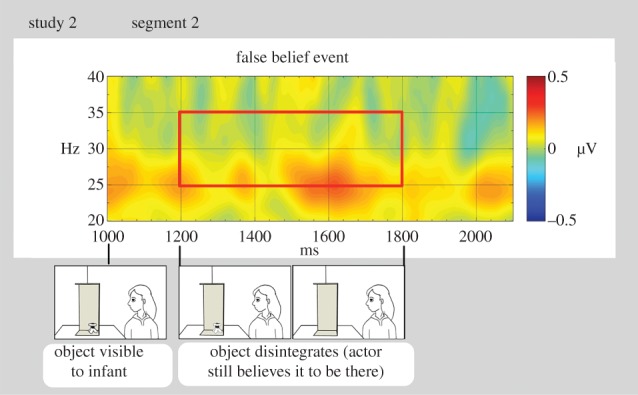
Time–frequency analysis of the average EEG during the false belief event at 10 electrodes over the left and right temporal cortex in study 2. The plot reflects mean activation difference between conditions; positive difference indicates higher activation in object occluded—false belief condition than in object absent—true belief. 1200 ms is the onset of the disintegration event and 1800 ms is the offset. The red rectangle indicates the time and frequency range over which statistics were computed.

Next, we entered the activation during the false belief segment of study 2 in a two-way ANOVA with condition (object occluded—false belief versus object absent—true belief) and hemisphere (left versus right) as within-subjects factors. There was a significant main effect of condition (*F*_1,14_ = 8.47, *p* = 0.01, partial *η*^2^ = 0.38) due to significantly higher activation in the object occluded—false belief (*M* *=* 0.07 µV, s.e. *=* 0.04 µV), compared with object absent—true belief condition (*M* *=* −0.01 µV, s.e. = 0.05 µV; figure 4). There was no main effect of hemisphere, and no interaction.

Finally, we analysed the late burst activation in the occlusion from actor segment in the left hemisphere to test whether our findings from study 1 were replicated. We analysed activation from the two studies with condition (object present versus object absent) as within-subjects factor and study (1 versus 2) as between-subjects factors. There was a significant main effect of condition (*F*_1,28_ = 7.97, *p* = 0.01, partial *η*^2^ = 0.22), due to higher activation in the object present (*M* *=* 0.07 µV, s.e. *=* 0.03 µV) than in object absent condition (*M* *=* −0.09 µV, s.e. = 0.04 µV), and no main effect of study (for detailed analysis in this late activation, see electronic supplementary material, additional analyses).

### (c) Discussion

The results of study 2 are consistent with the proposal that infants ascribe object representations to others not only when they attribute true beliefs, but also when they can attribute false beliefs to them. Similar to study 1, belief attribution here was based on visual perspective taking (infants had to encode that the object was not visible to the person). Crucially, in the false belief segment, when the object disintegrated and this was visible to the infant but not to the person, there was increased gamma-band activation, similarly to the occlusion events (occlusion from the infant or from the person).

These results suggest that infants encode that the other person continues to represent the object, despite evidence that prompts them to discard their own representation of the very same object. Because disintegration has been previously shown not to trigger sustained object representation [18], higher gamma activation during this event reflects that the infants sustained the object representation they had attributed to the actor (who falsely believed the object to be behind the occluder), even though this representation was in sharp conflict with the infants' own perception (as the object disintegrated). Thus, the infants must have encoded that the other person had seen the object being occluded, but did not see the disintegration, and hence the attributed object representation could not be discarded on her behalf, but had to be possibly refreshed and sustained further.

We see no obvious ways to explain the activation patterns we observed in study 1 and 2 in terms of simpler cognitive mechanisms that do not involve belief attributions. First, activation during occlusion from the actor only (occlusion from actor segments in both studies) could not be due to infants' own sustained representation because they continued to see the object during this event. Second, our results cannot be attributed to perceptual differences between the conditions (e.g. that the object was present in one condition but not in the other), because we subtracted the corresponding baseline activation from our data where this difference already existed, hence any activation difference due to this factor would have been thus subtracted from the time window of interest. Furthermore, results from the occlusion from actor segment in study 2 excluded the possibility that the gamma-band activation in the occlusion from actor segment was due to infants' expectation of occlusion from their own perspective, as no such occlusion followed.

Additionally, results from study 2 confirm the late burst of activation we found in study 1. This additional burst of activation therefore was present in both studies towards the end of the occlusion from actor segment. During this period that followed after the occlusion of the object from the actor, nothing was happening in the video. Therefore, this activation probably reflects computational processes that involve further processing of the earlier observed events, possibly related to keeping in mind the object representation attributed to the actor.

## 4. General discussion

The goal of this paper was to investigate whether young infants ascribe representations to others during tracking of what this other person sees, knows or believes, through using their own representational system that is otherwise used for encoding objects and events in the world. In study 1, we presented infants with scenes depicting a simple situation involving an object and actor, and events where the infants' or the other person's perceptual access to the object changed dynamically. In study 2, we constructed a case where this event could lead to a false belief about the presence of the object in the other person. We recorded event-related oscillatory activity during the observation of these events.

Earlier studies [18,19] found gamma-band oscillatory activity in infants for sustained object representation. We found similar gamma-band activations when an object became occluded from the infants' own (study 1, occlusion from infant) or someone else's perspective (studies 1 and 2, occlusion from actor), consistent with the possibility that there are shared underlying mechanisms for sustained object representations for the self and for the ones attributed to another person. Crucially, the activation found in response to object occlusion from the other person's perspective could only be explained by the enrolment of an object representation ascribed to her. This is supported by the fact that during this interval infants continued to perceive the object and therefore did not need to sustain the representation for themselves. Importantly, the same activation was observed in a false belief situation where, after being occluded from the actor, the infant saw the object disintegrating (study 2, false belief segment). Due to disintegration the object ceased to exist from the infant's point of view; therefore, EEG activation during this event is likely to be due to a sustained object representation on behalf of the actor. Together, the activations we found are indicative of the online processing of a representation that infants attribute to another person based on her earlier perceptual access, specifically in the form of a metarepresentation.

While we investigated here one kind of belief content (beliefs about the presence of objects), based on an approach arguing for shared mechanisms for infants' own representations and for attributed belief representations, other kinds of belief contents should also activate the corresponding cognitive systems in the observer's mind and be involved in operations on the ascribed belief representations. Recent evidence suggests that adults show an increased N400 effect when they can infer that another person will have difficulty integrating a sentence due to a semantically incongruous word (semantic violation) while they do not perceive it as semantic violation, similar to the cases when only they themselves perceive a semantic violation [20].

Our findings raise the question of how infants' primary representations would be separated from the representations ascribed to others. While this study does not directly address this question, we observed an additional burst of activation that accompanied only processing the object occlusion from the actor's perspective, in both studies. The fact that similar activation did not occur during the occlusion from the infant events suggests that it might reflect some further processing of ascribed representations, and could potentially play a role in distinguishing an ascribed representation from the infants' own reality representation.

The finding that the cognitive systems that are otherwise dedicated to representing objects are also involved in mentalizing processes points to the possibility that infants recruit cognitive systems from outside of a hypothesized ToM network [21] or ToM module [22] when representing others' beliefs. Yet we do not take such data to speak to the question that has repeatedly emerged with regard to ToM capacities, namely whether such reasoning is predominantly subserved by domain-general or domain-specific processes [23]. The gamma activations found in the ‘occlusion from actor’ events are most likely to signal sustaining an attributed representation of an object. This process relates to the encoding of the *content* of the actor's belief, in other terms to the formation of a metarepresentation of this belief content. However, as this is probably one of the first steps in the process of belief ascription [24], our findings leave open the possibility that in the further steps of belief processing such representations would serve as input to more specialized mindreading processes.

Metarepresentations involving mental states were argued to differ from other kinds of metarepresentations, for instance from those of public representations (such as utterances) or abstract representations, and there could be separate metarepresentational competencies for each, as a distinct evolved adaptation [13]. In line with this, behavioural evidence suggests that adults process belief representations distinctly from other metarepresentations that are not representations of mental states (such as vignettes describing an event) [25].

Together, our studies demonstrate that preverbal infants engage in encoding what others can and cannot see, and their consequential beliefs. By possessing such powerful representational capacities, infants are endowed with the ability to ascribe to others any representations they themselves can form, including representations that are in conflict with their own representation of reality. One might wonder whether these capacities are innate or are subject to change during development. While this is a question for future studies, it is possible that some basic ToM mechanisms have an innate basis, although they probably require some critical social experience.

Representing beliefs through forming metarepresentations of ascribed representations seems possible before the onset of language. The fact that language might not be necessary to form metarepresentations in belief reasoning raises the possibility that similar cognitive processes could be present in non-human animals. By the logic that cognitive systems responsible to represent the physical world might be ‘re-used’ to represent others' mental state contents, the question emerges whether non-human animals could in some situations metarepresent their conspecifics' representations. However, in the absence of empirical evidence, this question remains a task for future research.

## Supplementary Material

ESM kampis_parise_csibra_kovacs

## Data accessibility

For the time–frequency transformations, we used EEGLAB, an open-source toolbox for Matlab (http://www.mathworks.com/products/matlab/) available at http://sccn.ucsd.edu/eeglab, and the custom-made script collection WTools (available on request). The EEG dataset is available at http://datadryad.org/review?doi=doi:10.5061/dryad.r5p6q.
