# Supplementary material for: Neural signatures for sustaining object representations attributed to others in preverbal human infants
Source: Proc Biol Sci. 2015 Nov 22;282(1819):20151683. doi: 10.1098/rspb.2015.1683 (PMC4685805; doi:10.1098/rspb.2015.1683)
Supplement: ESM kampis_parise_csibra_kovacs [file rspb20151683supp1.pdf]

Electronic Supplementary Material for:

Neural signatures for sustaining object representations attributed to others in preverbal human infants

Dora Kampis, Eugenio Parise, Gergely Csibra, and Ágnes Melinda Kovács

[a]Department of Cognitive Science, Central European University, Budapest, Hungary 1015

[b]Flyde College, Lancaster University, Lancaster, UK LA1 4YF

[c]Department of Psychological Sciences, Birkbeck, University of London, London, UK WC1E 7HX

I. SI Materials and Methods

Study 1.

Participants. 15 infants included in the analysis, and 24 additional infants were tested but not included in the analysis due to fuzziness (7), extensive body movements (4), insufficient number of trials (10), noise in the recording (2), or maternal interference (1).

Stimuli and Procedure. The same testing apparatus was used in both studies. Infants sat in a dimly lit soundproof room, 70 cm from a CRT monitor (100 Hz refresh rate), on their parent's lap. The parents were instructed to not to communicate with the infant. Infants watched a maximum total number of 60 trials. If infants were not attending to the screen and could not be reoriented, a short brake was included. Videos of the infants were recorded during the presentation of the stimuli, in order to assess their looking behaviour.

The videos displayed a female actor and a rotating box that could contain 5 different objects varying in colour and shape. Altogether 10 different videos were used, with 5 objects in each condition (Object Present - Occlusion vs. Object Absent - Occlusion), which were presented in a pseudo-random order with no more than 3 consecutive trials involving the same object or condition.

In each trial, a colourful fixation stimulus appeared on the screen (the fixation stimulus changed after every 10 trials), and a short sound was played before each trial to orient the infant's attention to the screen. The duration of the fixation stimulus varied randomly between 600-800 ms. If the infant did not orient to the screen, a looming spiral appeared until they looked at the screen. During the trials, the videos were shown, each of them lasting 5300 to 5700 ms, depending on the length of the jittering periods. First infants saw the backside of the box and the person facing the infant for 200ms. Then the box rotated (for a duration of 600ms; first rotation), and the content of it (the object) was revealed. Then after 200 ms of still image the person turned her head (for 600ms) and looked at the object. Afterwards, depending on the condition, the object either gradually disintegrated or it remained there (for 600ms). This was followed by a still image for a jittered period between 300-500 ms, the last 200ms of which served as baseline period. Then the box started to rotate (for 600ms; second rotation), gradually hiding the object (Object Present - Occlusion condition) or the place where it was before disintegrating (Object Absent - Occlusion condition) from the eyes of the actor. After a still period of 700-900 ms the rotation continued (for 600 ms; third rotation), hiding the object (or the place where it had been) from the eyes of the infant. Finally, the image with the box turned away remained still for 800ms. The interval between trials lasted on average 1100 ms (randomized between 1000-1200 ms). In this interval first a blank screen, then the fixation stimulus was shown. The length of the still periods during trials, and the fixation cross between trials were varied to avoid anticipatory effects. Note that according to the 'Advisory Notice' communicated by EGI (Electrical

Geodesics Inc., Eugene, OR, USA) to their costumers on August 29, 2014, the EEG recorded at a 500 Hz sampling rate with the GES 300 amplifier were delayed by 18 ms compared to the time-stamps due to their implementation of anti-aliasing filters. This delay resulted in a shift of the actual time windows of our measurement from 550-650 ms to 532-632 ms.

EEG analysis. Segments that were judged as not attended based on the video recording were excluded. Artefact detection and removal was performed using both automatic (by the Net Station tool, NetStation 4.4, Electrical Geodesics, Inc.), and manual methods. Segments with more than 20 channels (>~15% of the channels) containing artefacts (eye-movements, blinking, electrical noise) were excluded from the analysis. Bad channels were interpolated in the remaining segments. Infants contributed a mean number of 30 trials to the Occlusion from Actor segment (15/condition), and 24 to the Occlusion from Infant segment (12/condition). The lower number of average trials in Occlusion from Infant was due to this segment being later in the trial, and infants became less attentive, resulting in a larger amount of artefacts. Infants who did not contribute a minimum of 10 trials per condition for the Occlusion from Actor segment (or 5 trials for the Occlusion from Infant segment) were excluded from the analysis.

The retained segments were imported into Matlab® using the toolbox EEGLAB (v. 9.0.5.6b) and re-referenced to the average reference. Time-frequency transformations were computed using EEGLAB and the custom-made script collection WTools (available at request) using continuous

complex Morlet wavelets at each 1 Hz frequency bin between 5 and 60 Hz. An additional 400 ms of recording was left both at the beginning and at the end of the segments for the distortion caused by the wavelet transformation, which intervals were not included in the final analysis.

Transformed data were baseline-corrected to the average activity during the 200-ms-long baseline epoch. This epoch was the 200 ms recording preceding the rotation of the occluder in the Occlusion from Actor segment. In the Occlusion from Infant segment we used an epoch that roughly matched the baseline period in the first segment: a 200-ms-long interval ending 1500 ms before the onset of Occlusion from Infant (approximate comparison to baseline of Occlusion from Actor is due to a jitter period introduced between the two segments of a length varying between 100 and 300 ms). We selected this baseline based on the consideration that during this period the differences between conditions in terms of presence or absence of the object were already present, hence any possible difference in activation later cannot be simply due to the fact that in one condition infants saw an object during the crucial events whereas in the other they did not.

Study 2. *Participants*. 15 infants included in the analysis, and further 30 infants were tested but not included in the analysis due to fuzziness (7), extensive body movements (10), insufficient number of trials (8), noise in the recording (3), experimental error (1), or maternal interference (1).

Stimuli and Procedure. The procedure of the experiment, as well as the stimuli with regard to the parameters of the intervals between trials, the fixation crosses and orientation sounds, and the re-orienting spiral were identical to those in Study1.

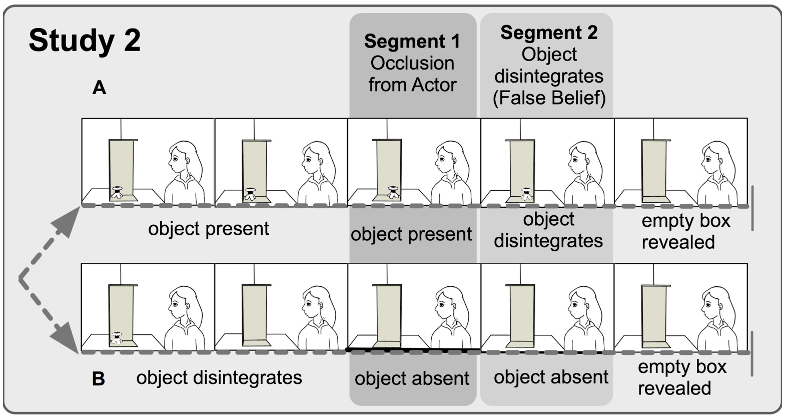

Figure 1. Schematic illustrations of the critical events in Study 2.

Each trial began with the box oriented towards the person and the object visible. After 200 ms of still image the actor turned her head (for 600 ms) and looked at the object. Then after 100 ms, depending on the condition the object either gradually disintegrated over 600 ms (Object Absent – True Belief condition) or it remained there (Object Occluded – False Belief condition). This was followed by a still image for 300-500 ms, the last 200 ms of which served as baseline period. Then the box started to rotate (for 600 ms), gradually hiding the object (or the place where it was before) from the eyes of the actor. After a still period of 600 ms in the Object Occluded – False Belief condition the object disintegrated during the next 600 ms; while in the Object Absent – True Belief condition the occluded space remained empty during this time. This was followed by a still period of 300-500 ms, during which the box

was already empty in both conditions. Finally, the empty box rotated back in 600 ms, and then stood still for another 600 ms.

Infants contributed a mean number of 29.5 trials in the Occlusion from Actor segment (on average 14.8/condition), and 27.3 in the False Belief segment (13.6/condition) in Study 2.

2. SI Additional Analyses – late burst activation

In addition to analysing activation in our predicted time windows, after visual inspection we analysed activation in the Occlusion from Actor segment at a later time point, starting at 1000 ms after the onset of Segment 1. This interval was not part of our original time window of interest (time of full occlusion from the actor) as it was 400 ms after the object was completely occluded from the actor and there were no events happening at this time. Results are shown in Figure 1. We analysed this activation for the 1000-1100 ms period in a repeated measure ANOVA with Condition (Object Present - Occlusion vs. Object Absent - Occlusion) and Hemisphere (left vs. right) as within-subjects factors. We found a significant interaction between Condition and Hemisphere ($F(1,14) = 5.37$, $p = .036$, $\eta^2 = .277$). We found a marginally significant effect of Condition in the left hemisphere ($t(14)= -2.07$, $p = .057$, $r^2 = .23$) with higher activation in the Object Present - Occlusion condition than in the Object Absent - Occlusion condition ($M = 0.09$ μV, $SE = .05$ μV, and $M = -0.11$ μV, $SE = .07$ μV, respectively). There was no effect of Condition in the

right hemisphere ($t(14) = -0.42$, $p = .68$). A similar analysis did not yield any significant main effects or interactions in Segment 2. Since this late activation burst in Occlusion from Actor segment was not expected, we intended to replicate it to assess the robustness of the finding in Study 2.

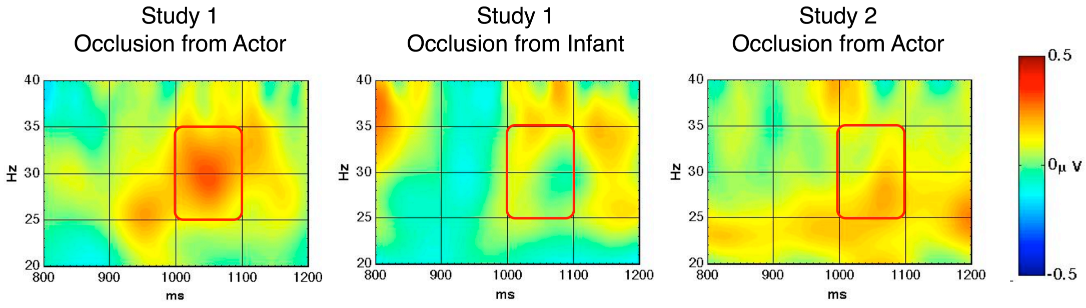

Figure 2. Time-frequency difference plots depicting average gamma-band oscillatory activation over the left and right posterior temporal cortex showing the late burst activation. Plots show 800-1200 ms after segment onset and reflect mean activation difference between conditions; positive difference indicates higher activation in Object Present condition than in Object Absent condition. Red rectangles indicate the time and frequency range over which statistics were computed.

After performing Study 2 we analysed the late burst activation from the two studies in the Occlusion from Actor segment in a 3-way mixed ANOVA with Condition (Object Present vs. Object Absent) and Hemisphere (Left vs. Right) as within-subjects, and Study as between-subjects factor. There was a significant main effect of Condition ($F(1,28) = 5.28$, $p = .029$, *partial* $\eta^2 = .16$), due to significantly higher activation in the Object Present condition than in Object Absent condition ($M = 0.05$ μV, $SE = 0.02$ μV, and $M = -0.05$ μV, $SE = 0.03$ μV, respectively). There was also a significant Condition*Hemisphere interaction ($F(1,28) = 4.82$, $p = .037$, *partial* $\eta^2 = .15$). We analysed activation

separately in the two hemispheres in a two-way mixed ANOVA with Condition (Object Present vs. Object Absent) as within-subjects factor and Study (1 vs. 2) as a between-subjects factor. On the left ahemisphere there was a significant main effect of Condition ($F(1,28) = 7.97$, $p = .01$, *partial* $\eta^2 = .22$), due to higher activation in the Object Present ($M = 0.07$ μV, $SE = .03$ μV), than in Object Absent condition ($M = -0.09$ μV, $SE = .04$ μV). Similar analysis on the right hemisphere did not yield any significant main effects or interactions.

This additional burst of activation therefore was present in both studies towards the end of the Occlusion from Actor segment. At this time nothing was happening in the video, therefore this activation likely reflects computational processes that involve further processing of the earlier observed events. Since this late activation accompanied only processing the object occlusion from the other person, it might reflect some further processing or tagging the sustained representation as ascribed to the other person, hence possibly playing a role in distinguishing an ascribed representation from the infants' own reality representation.
